# Side Flow Effect on Surface Generation in Nano Cutting

**DOI:** 10.1186/s11671-017-2136-3

**Published:** 2017-05-19

**Authors:** Feifei Xu, Fengzhou Fang, Xiaodong Zhang

**Affiliations:** 1 State Key Laboratory of Precision Measuring Technology and Instruments, Centre of MicroNano Manufacturing Technology, Tianjin University, Tianjin, 300072 China; 2 Institute of Mechanical Manufacturing Technology, China Academy of Engineering Physics, Sichuan, 621900 China; 3 School of Mechanical and Materials Engineering, MNMT-Dublin, University College Dublin, Dublin, Ireland

**Keywords:** Nano cutting, Side flow, Surface generation, Plastic deformation, Cutting mechanism

## Abstract

The side flow of material in nano cutting is one of the most important factors that deteriorate the machined surface quality. The effects of the crystallographic orientation, feed, and the cutting tool geometry, including tool edge radius, rake angle and inclination angle, on the side flow are investigated employing molecular dynamics simulation. The results show that the stagnation region is formed in front of tool edge and it is characterized by the stagnation radius *R*_*s*_ and stagnation height *h*_*s*_. The side flow is formed because the material at or under the stagnation region is extruded by the tool edge to flow to the side of the tool edge. Higher stagnation height would increase the size of the side flow. The anisotropic nature of the material which partly determines the stagnation region also influences the side flow due to the different deformation mechanism under the action of the tool edge. At different cutting directions, the size of the side flow has a great difference which would finally affect the machined surface quality. The cutting directions of {100} < 011>, {110} < 001>, and {110} < 1-10 > are beneficial to obtain a better surface quality with small side flow. Besides that, the side flow could be suppressed by reducing the feed and optimizing the cutting tool geometry. Cutting tool with small edge radius, large positive rake angle, and inclination angle would decrease the side flow and consequently improve the machined surface quality.

## Background

Ultra-precision cutting is one of the most common methods in realizing the nanometric surface roughness and sub-micrometric form accuracy. Based on the assumption that the workpiece material is removed ideally and the machined surface texture is the replication of tool nose profile, the surface roughness could attain nanometric even sub-nanometric scale, if simply decreasing the feed and increasing the tool nose radius. However, it always deviates from its ideal value due to many factors, such as, material properties of workpiece [1–4] and cutting tool geometry [5–7]. The side flow of workpiece material, which is a result of the interactions between cutting tool and workpiece material, is considered to be an important role in causing the deviation [8]. It is the plastic deformation of workpiece material at a direction opposite to the feed direction [9]. Two mechanisms are proposed to describe the formation of material side flow [7]. The first one is the squeeze between the tool flank face and the machined surface especially when the uncut chip thickness (UCT) is less than the minimum uncut chip thickness. The second one is due to the trailing edge notch. The material is pressed aside under high temperature and pressure. Material side flow has been modeled by 3D thermo elasto-viscoplastic finite element method and found that more side flow is generated with higher nose radius and lower feed [10]. The side flow of material has been taken into account to predict the machined surface roughness [8, 11].

In nano cutting, the ever-reduced UCT making the material removal at nanoscale which is smaller than the material grain size causing the significant influence of the size effects [12] and material removal mechanism [13–15]. The anisotropic nature of single crystal materials would appear in the nano cutting, even the machined materials are polycrystalline [16]. Lee et al. investigated the anisotropy of surface roughness on three different crystal planes in cutting process [3]. The difference of machined surface roughness is explained by the different amount of recovery induced by the anisotropic Yong’s modulus. To et al. found that {100} plane of single crystal aluminum could attain a best machined surface roughness [2]. The anisotropy of single crystal 3C-SiC during nano-cutting has also been found by Goel et al. using molecular dynamics (MD) simulation [1]. However, the influence of anisotropic nature of single crystal material on side flow has not been deeply investigated by far, which plays an important role in determining the machined surface quality.

Besides that, when the UCT is comparable to the cutting tool edge radius, the effect of tool edge on machined surface quality could no longer be neglected. For instance, the increased tool edge radius would increase the surface roughness [17], and the spring back of the machined material affected by the cutting tool edge is thought to be responsible of the machined surface roughness [18]. At the tool edge, there is a region or point where the material tends to separate [19, 20]. The point is thought to be the stagnation point [14, 15, 21], and the region is the stagnation region where the material flow velocity is almost zero [22]. The material above the stagnation point or region is removed to form the chip. The material below them is pressed down to the flank face of cutting tool to form the machined surface. The amount of pressed material which is determined by the tool edge radius, is responsible of the side flow and further affects the generated surface quality. However, the influence of cutting tool geometry on side flow still lacks deep investigations in nano-cutting.

In this study, the effects of the crystallographic orientation and the cutting tool geometry, including tool edge radius, rake angle and inclination angle, on the side flow are investigated employing MD simulation. This study contributes to a better understanding of the surface generation for single crystal materials and polycrystalline materials in nano-cutting.

## Methods

**Fig. 1:**
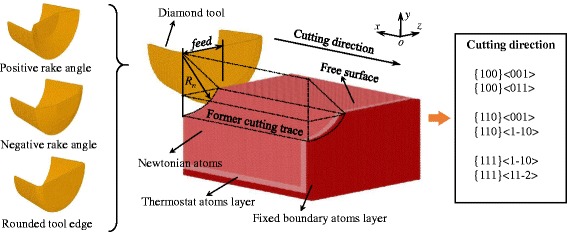
Schematic description of nano cutting model with three kinds of cutting tools and six cutting directions

MD simulation is employed to investigate the side flow of aluminum during nano cutting. As shown in Fig. 1, the MD simulation model consists of a rigid diamond tool and an aluminum workpiece. The tool edge radius *r*_*β*_ changes from 0 to 7.5 nm and the nose radius *R*_*n*_ stays constant at 15 nm. The rake angle of the cutting tool changes at the range of −30° to 30° and the clearance angle is 12.5°. In addition, the inclination angle of the cutting tool changes from −8° to 20°. The size of workpiece is 45 nm × 38 nm × 20 nm and it containing about 2,000,000 atoms. Part of workpiece is cut off by a cylinder with radius of 15 nm which is the tool nose radius *R*_*n*_ to simulate the former cutting trace left on the workpiece surface. Therefore, the side flow on the nano cutting or turning processes could be investigated efficiently. Atoms of workpiece are defined as three parts: boundary layer, thermostat layer and Newtonian layer. Atoms in boundary layer are fixed at space to prevent the unexpected movement under the action of cutting force. The thermostat layer adjacent to it is kept at a constant temperature of 293 K to imitate the heat dissipation in nano cutting. The rest atoms that would move under the cutting of tool are in the Newtonian layer obeying the Newton’s law.

Six cutting directions, including {100} < 001>, {100} < 011>, {110} < 001>, {110} < 1-10>, {111} < 1-10 > and {111} < 11-2>, are considered in investigating the effect of crystallographic orientation on side flow in nano cutting. Depth of cut is set to 5 nm and the feed is change from 1.5 to 15 nm. Therefore, the UCT along the cutting tool edge changes with the different combination of feed and depth of cut. The cutting speed is 100 m/s at the negative x-direction. The cutting distance of the model is about 40 nm. Initial temperature of the cutting model is equal to the constant temperature in thermostat layer.

The interaction among the aluminum atoms is described by the embedded atom method (EAM) potential [23]. The tool energy *E* is

1 $$ E={\displaystyle \sum_i{F}_i\left({\rho}_i\right)}+\frac{1}{2}{\displaystyle \sum_{i, j, j\ne i}{\phi}_{i, j}\left({r}_{i j}\right)} $$

where *F*_*i*_(*ρ*_*i*_) is the embedding energy to embed atom *i* into the electron density *ρ*_*i*_, and *ϕ*_*i*,*j*_(*r*_*ij*_) is the pair potential energy between atoms *i* and *j*. The electron density *ρ*_*i*_ can be calculated by the following form

2 $$ {\rho}_i={\displaystyle \sum_{j, j\ne i}{f}_j\left({r}_{i j}\right)} $$

where *f*_*j*_(*r*_*ij*_) is the electron density casing by atom *j* which has a distance of *r*_*ij*_ to the location of atom *i*.

The interaction between the carbons atoms is ignored due to the diamond is much harder than aluminum and the diamond tool is thought as rigid. The interaction between the rigid diamond tool and aluminum atoms is depicted by the Morse potential:

3 $$ E={D}_0\left[{e}^{-2\alpha \left( r-{r}_0\right)}-2{e}^{-\alpha \left( r-{r}_0\right)}\right] $$

where *E* is the pair potential energy, *D*_0_ is the cohesion energy, *α* is a constant determined by material properties, *r*_0_ is the distance at equilibrium and *r* is the distance between two atoms.

The MD simulation is based on the LAMMPS and the microstructural evolution of the workpiece under cutting process is analyzed based on common neighbor analysis with software OVITO. The displacement vectors of the workpiece atoms are analyzed to reveal the material side flow mechanism in nano cutting.

## Results and Discussion

### Stagnation Region and Cutting-Induced Side Flow

**Fig. 2:**
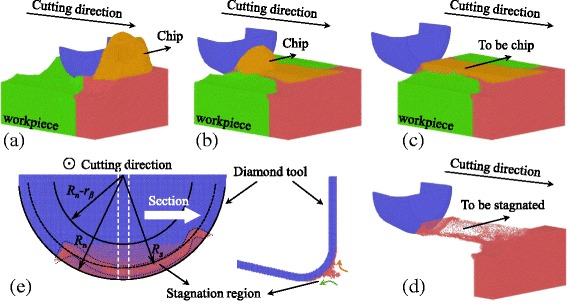
Evolution of nano cutting process at cutting direction of {100} < 001>, tool edge radius of 5 nm and cutting distance of (**a**) 40 nm, (**b**) 10 nm, (**c**) 0 nm, and (**d**) the atoms to be stagnated, (**e**) forms the stagnation region on the cutting tool edge

Figure 2a–c are snapshots of the MD simulation with the cutting distance of 40, 10, and 0 nm. Atoms which are removed to form the chip or pressed down to form the machined surface are assigned with yellow and green colors. As shown in Fig. 2a, atoms in front of tool rake face and above the workpiece free surface are considered to form the chip and the atoms beneath the tool flank face are considered to form the machined surface. Then the atoms motion in the nano cutting process and the boundary (atoms in red color) could be obviously observed. When the yellow and green atoms are not displayed, as shown in Fig. 2d, atoms in the red boundary are stagnated or trapped by the cutting tool edge. More time and cutting distances the red atoms should take to determine whether to be a part of chip or machined surface. The stagnated atoms form the stagnation region on the cutting tool edge as shown in Fig. 2e. The shape of the stagnation region could be approximate to an arc with the center locating at the center of tool nose and its radius denotes as *R*_*s*_. The section of the stagnation region has a triangular shape and more atoms would join in and maintain the region with the cutting process. Atoms above the stagnation region tend to form the chip and atoms below the stagnation region are pressed to form the machined surface.

**Fig. 3:**
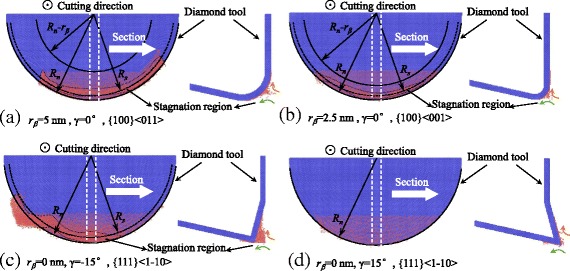
Stagnation region on the cutting tool edges

The stagnation region on the different tool edge is shown in Fig. 3. The stagnation radius *R*_*s*_ for {100} < 011 > cutting direction is larger than that of {100} < 001 > cutting direction in Fig. 2e, which means less material is pressed and flow to the flank face of the cutting tool to form the machined surface. When cutting with a tool edge radius of 2.5 nm (see Fig. 3b), the stagnation radius *R*_*s*_ is larger than that with 5 nm tool edge radius (see Fig. 2e). The sharp tool edge with a negative rake face entraps a large number of atoms forming a large stagnation region at its edge, especially at its side, as shown in Fig. 3c. However, when cutting the material by a sharp tool edge with a positive rake angle, almost no atoms are entrapped and no stagnation region forms at the tool edge (see Fig. 3d).

**Fig. 4:**
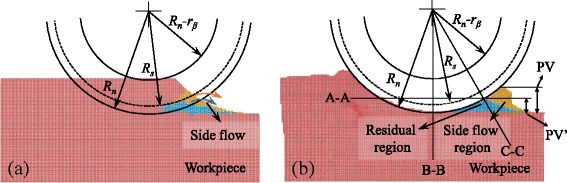
Atoms tend to form the side flow region (*yellow*) and residual region (*blue*), (**a**) before and (**b**) after nano-cutting in {100} < 001 > cutting direction

In nano-cutting, the workpiece material is subjected to the action of tool edge to cause the material to flow to the side, as shown in Fig. 4. The yellow region in Fig. 4b is the side flow material after the cutting process and the blue region is supposed to be the ideal material that would not be removed left on the machined surface. These two regions are called as the side flow region and the residual region, respectively. Therefore, the surface roughness, i.e., the PV (peak-to-valley) value generated on the machined surface is larger than the ideal value PV’, due to the formation of side flow. Figure 4a is the distribution of the yellow and blue atoms in the workpiece before cutting. A larger part of them are at or under the stagnation region which is characterized by the stagnation radius *R*_*s*_. Then, in the cutting process, they are extruded by the tool edge to form the side flow at the side of the tool edge. The position, shape and size of the stagnation region would influence the material flow under action of tool edge and it is determined by the material properties and tool edge geometry. All the factors mentioned are discussed in the following sections.

### Effect of Cutting Direction

**Fig. 5:**
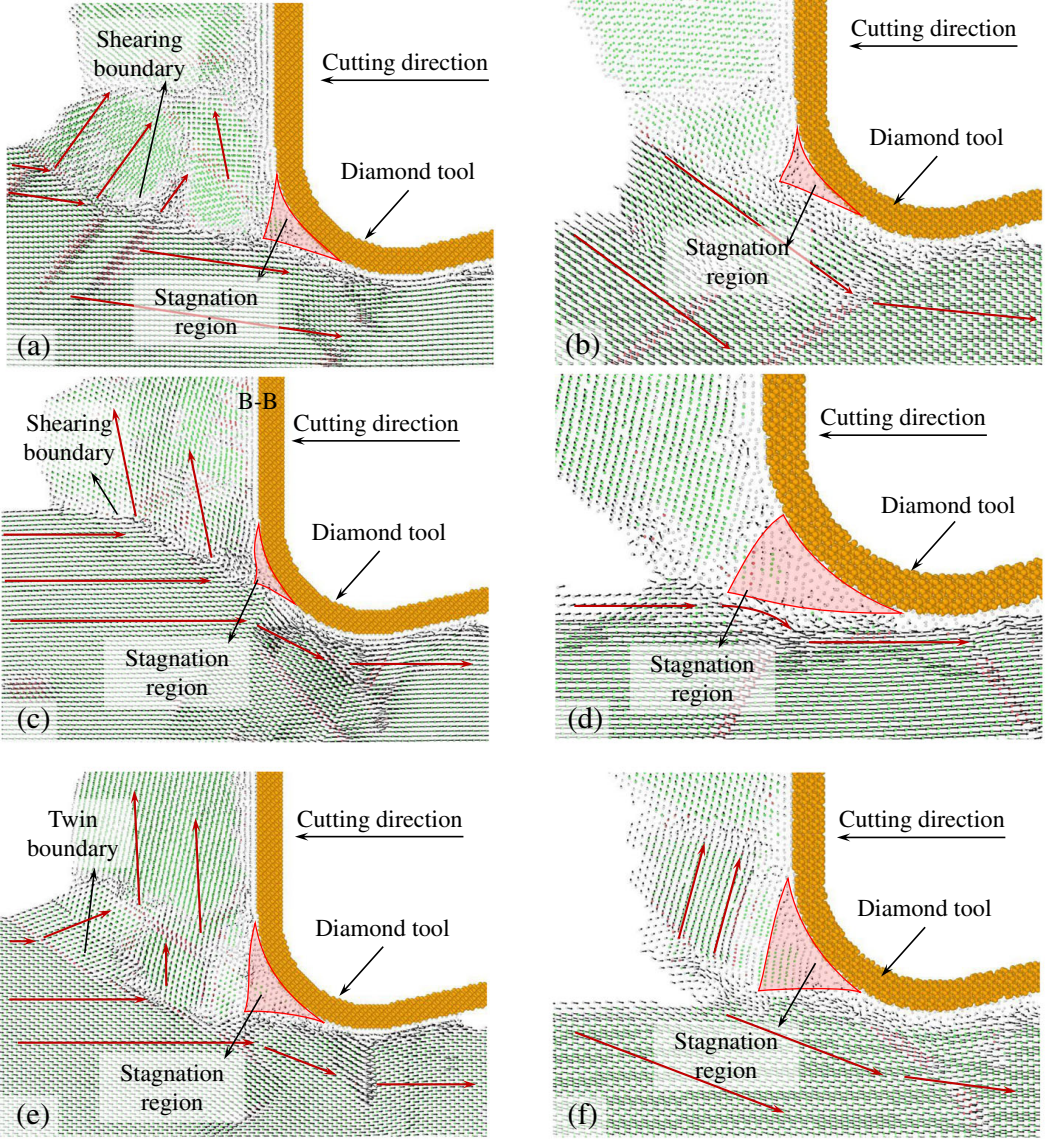
Displacement vectors at cutting direction of {100} < 001>, and slice plane of (**a**) B-B, (**b**) C-C; cutting direction of {100} < 011>, and slice plane of (**c**) B-B, (**d**) C-C; cutting direction of {110} < 001>, and slice plane of (**e**) B-B, (**f**) C-C

Figure 5 is the displacement vector at the plane B-B and C-C (see Fig. 4b) sliced with the thickness of 1 nm. Their cutting directions are {100} < 001>, {100} < 011 > and {110} < 001 > respectively. The displacement vector of atoms, to some extent, represents the material flow under the action of the cutting tool. At the plane B-B, as shown in Fig. 5a, c, and e, in which the UCT of the tool edge is equal to the depth of cut, the displacement vectors of workpiece atoms abruptly change at a boundary. The boundary is where the plastic deformation occurs. The boundary expands from the tip of the stagnation region which is formed in front of the cutting tool edge. The included angle between the boundary and the cutting direction is the shearing angle. The shearing angle of {100} < 001 > cutting direction is smaller than that of {100} < 011 > cutting direction at the snapshots. The shearing angle of the {110} < 001 > cutting direction is the largest because it is the twin boundary. The shearing angle of it is about 35°.

**Fig. 6:**
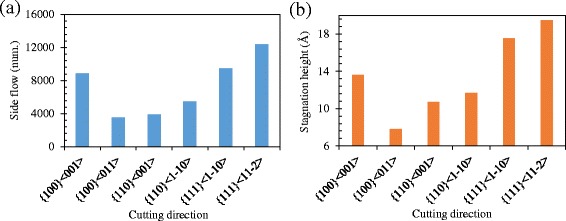
**a** Number of atoms flow to the side of tool edge and **b** stagnation height at the side of tool edge at different cutting directions

At the plane C-C, as shown in Fig. 5b, d, and f, it is the displacement vector of atoms when the UCT is almost zero, and it is where the side flow tends to occur. At the cutting direction of {100} < 001>, a large number of atoms are extruded by the tool edge to flow to the flank face of the cutting tool forming the side flow on the machined surface. However, at the cutting direction of {100} < 011 > and {110} < 001>, a small amount of atoms are extruded by the tool edge. Therefore, smaller side flow forms on the machined surface. As shown in Fig. 6, the size of side flow seems to relate to the stagnation radius *R*_*s*_ on the cutting edge. The stagnation height *h*_*s*_ which is calculated according to the equation (*R*_*n*_ − *R*_*s*_), is used to characterize the position of the stagnation region at the tool edge. As shown in Fig. 6(b), the stagnation height of the {100} < 001 > cutting direction is higher than that of the {100} < 011 > and {110} < 001 > cutting directions, which causes a larger side flow. The number of atoms in the side flow region (yellow region in Fig. 4b) which quantitatively represents the size of the side flow material is counted and shown in Fig. 6a including all the cutting directions. The side flow is larger for the cutting direction of {100} < 001>, {111} < 1-10 > and {111} < 11-2 > than that of the rest three cutting directions. And it is consistent with the stagnation height *h*_*s*_ at each cutting direction, as shown in Fig. 6b.

**Fig. 7:**
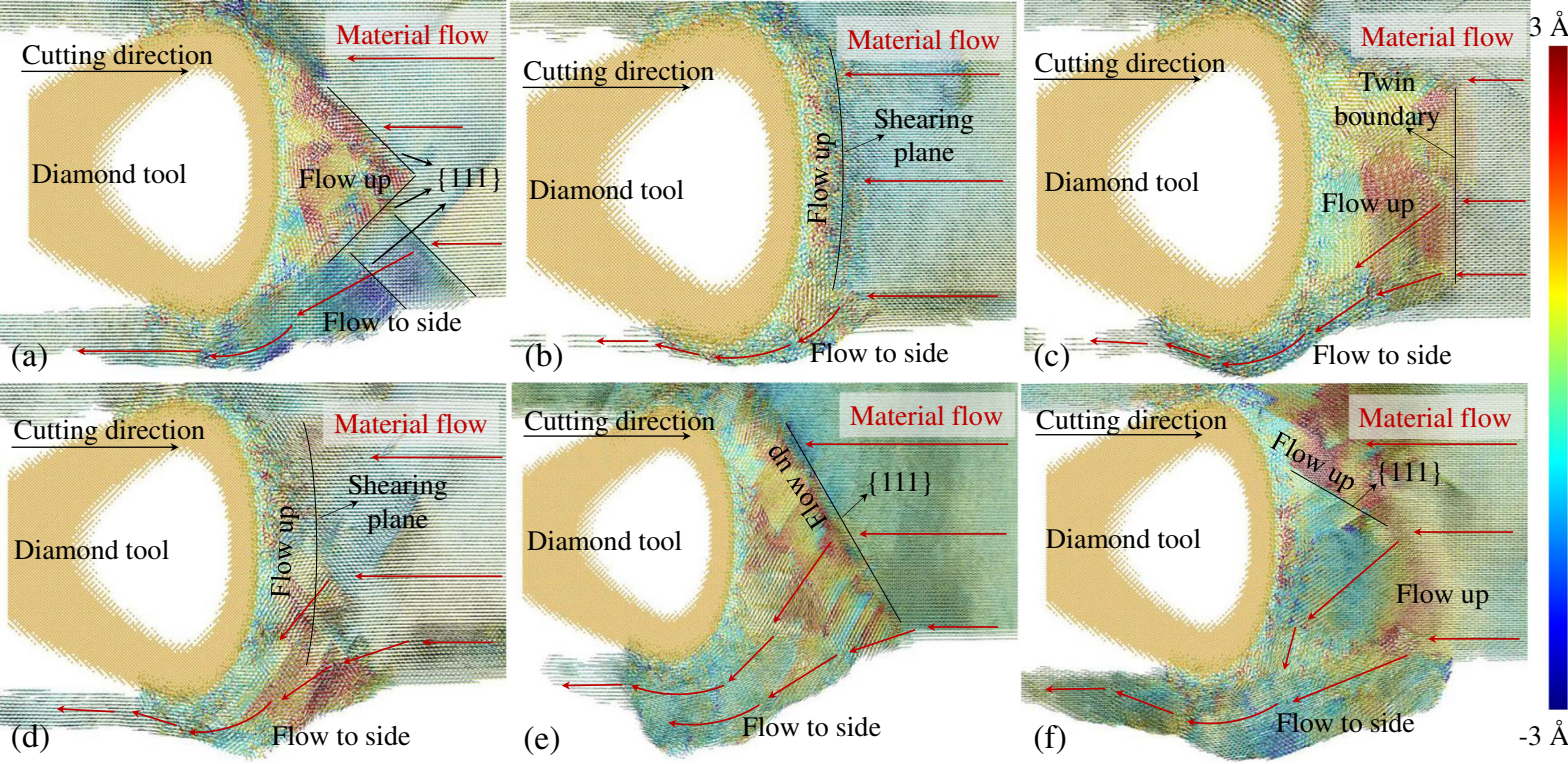
Displacement vector plots in cutting direction of (**a**) {100} < 001>, (**b**) {100} < 011>, (**c**) {110} < 001>, (**d**) {110} < 1–10>, (**e**) {111} < 1–10 > and (**f**) {111} < 11–2>

Furthermore, the side flow is influenced by the anisotropic nature of single crystal materials. At different cutting directions, the material deforms in different ways under the action of the tool edge. To exhibit the material deformation and side flow in different cutting directions, the atoms displacement vector at the plane A-A (see Fig. 4b) sliced with a thickness of 1 nm is displayed in Fig. 7. The atoms in the figures are colored with different colors according to the displacement at the y direction. Therefore, the atoms displacement vector in the space could be fully displayed. With the cutting direction of {100} < 001>, the material in front of the cutting edge flows up at a triangular region which is bounded by the {111} planes and the cutting edge, as shown in Fig. 7a. Out of the triangular region, large part of material flows to the side of the tool edge. And then the side flow which is relatively larger than the cutting direction of {100} < 011>, {110} < 001>, and {110} < 1–10>, is formed when the material flows to the flank face of the cutting tool. Similar results are obtained when cutting on the {111} plane which is a close-packed plane, a large number of atoms flow to the side of tool edge, as shown in Fig. 7e, f. It is because the material is easy to slip on the close-packed plane {111} making large part of material flow to the side of the tool edge.

When cutting with the {100} < 011 > direction whose side flow is the smallest, atoms do not flow to the side until a small amount of atoms reach the shearing plane. The flow up region in front of the cutting tool is bounded by the shearing plane which expands from the tip of the stagnation region as shown in Fig. 5c. Similar results are obtained when cutting with the direction of {110} < 1–10>. But more material flows to the side of the cutting edge forming the relatively larger side flow on the machined surface. With the cutting direction of {110} < 001>, the shearing plane is the twin boundary, as shown in Fig. 5e, which expands under the tool edge. Therefore, large part of material flow up in front of the tool edge and small part of material flow to the side of the tool edge. Thus it can be seen that the side flow is influenced by the anisotropic nature of the material. At different cutting directions, the size of the side flow has great difference which would finally affect the machined surface quality. Therefore, the cutting directions of {100} < 011>, {110} < 001>, and {110} < 1–10 > are beneficial to obtain a better surface quality. For further increasing the machined surface quality, the feed and the tool geometry could be optimized which are discussed in the next sections.

### Effect of Feed

**Fig. 8:**
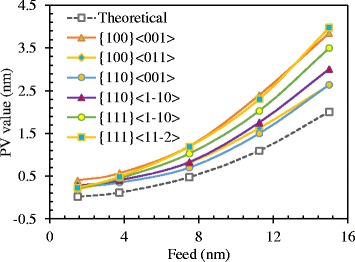
PV generated at different cutting directions

The effect of feed on the side flow and the material removal mechanism is investigated by changing the feed from 1.5 to 15 nm. The results shown in Fig. 8 are the theoretical and simulated PV value obtained according to Fig. 4. At all different cutting directions, the simulated PV value is larger than the theoretical value and decreases with the reduced feed. When the feed is larger than 8 nm, the PV value of {100} < 011 > and {110} < 001 > cutting directions is the smallest. And the largest PV value is obtained at {100} < 001 > and {111} < 11–2 > cutting directions. The PV value of the rest cutting directions is in the middle of them and the value of {111} < 1–10 > cutting direction is relatively larger that that of {110} < 1–10 > cutting direction. It is consistent with the number of atoms in the side flow region shown in Fig. 4a.

**Fig. 9:**
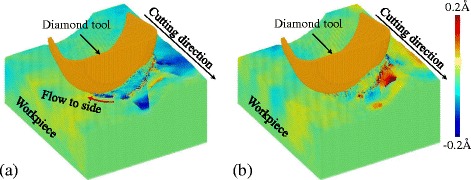
Displacement of atoms at (**a**) x-direction and (**b**) y-direction at cutting direction of {111} < 11–2 > and feed of 1.5 nm

**Fig. 10:**
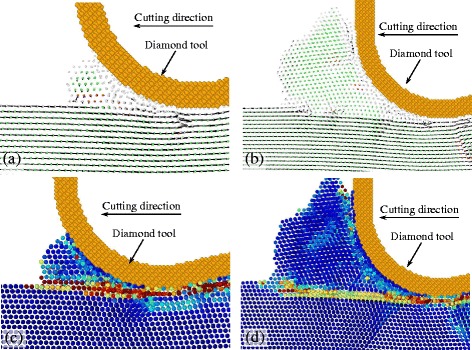
Displacement vector of atoms at the plane B-B, cutting direction of {111} < 11–2 > and the feed of (**a**) 1.5 nm and (**b**) 3.75 nm; (**c**) and (**d**) corresponding shear strain

When the feed is smaller than 8 nm, the PV value of all the cutting directions is close to each other. Minimum PV value is not found at which the PV value would increase when further decreases the feed [24]. It is because the cutting distance used in these simulations is small making the side flow increase slowly. As shown in Fig. 9 is the displacement of atoms in x-direction and y-direction at the feed of 1.5 nm. The atoms in front of the diamond tool tend to flow to the side and almost no atoms flow up along the rake face of the cutting tool. If the cutting distance is long enough, more atoms would be extruded causing the size of the side flow increases. The displacement vector of atoms at the plane B-B is shown in Fig. 10a, b. When the feed is 1.5 nm, the cutting tool is rubbing on the machined surface and almost no atoms are removed. When the feed is 3.76 nm, stagnation region does not form in front of tool edge and no shearing plane expands from the stagnation region tip, making the material extruded away from the bottom of the cutting edge and forming as chip. In this condition, the material is removed in extrusion mechanism [13–15]. Figure 10c, d is the corresponding shear strain distribution of Fig. 10a, b. The results show that when in the rubbing and extrusion mechanism, the primary deformation zone expands from the bottom of the cutting tool edge and just merges with the tertiary deformation zone. In rubbing and extrusion mechanism, the strain zone almost parallels to the cutting direction. The differences between the extrusion and rubbing mechanism are that the strain happens at surface or subsurface of the workpiece material.

### Effect of Tool Edge Radius

**Fig. 11:**
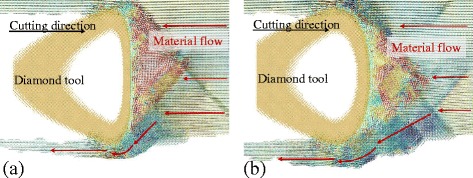
Displacement vector plots at tool edge radius of (**a**) 2.5 nm and (**b**) 5 nm in cutting direction of {100} < 001>

**Fig. 12:**
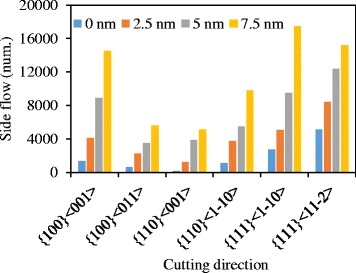
Number of atoms flow to the side of tool edge with different tool edge radius *r*_*β*_

It is known that higher stagnation height would increase the size of side flow. The tool edge radius would influence the stagnation height and further affect the side flow. Comparing the Fig. 2e and Fig. 3b, the separation height of the 2.5 nm tool edge radius is smaller than that of the 5 nm tool edge radius. The displacement vector plots of the cutting processes with tool edge radius of 2.5 nm and 5 nm are shown in Fig. 11 (at the same condition as the Fig. 7). More material is flow to the side of the tool edge forming the side flow on the machined surface. The number of atoms in the side flow region is counted and the results are displayed in Fig. 12. The results show that the sharper tool would decreases the size of side flow for all the six cutting directions.

In nano-cutting, large part of material is under the action of the tool edge whose effective rake angle is always negative regardless of the nominal rake angle is positive or not [14, 15, 25]. When the tool edge radius increases, more material is under the action of negative rake angle making the increase of side flow. Detailed discussion about the effects of the rake angle on the side flow in nano cutting is described in next section.

### Effects of Rake Angle

**Fig. 13:**
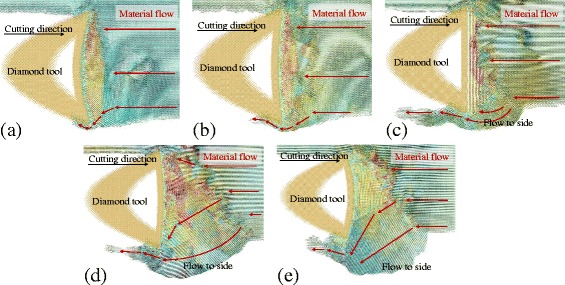
Displacement vector plots at rake angle of (**a**) 30°, (**b**) 15°, (**c**) 0°, (**d**) −15°, (**e**) −30°

**Fig. 14:**
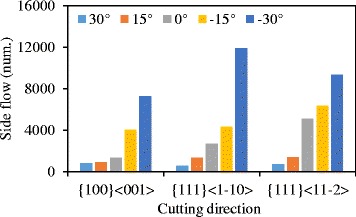
Number of atoms flow to the side of tool edge with different rake angle

In order to eliminate the influence of tool edge and only investigate the influence of rake angle on the side flow, the cutting tool with sharp edge and different rake angle, including 30°, 15°, 0°, −15°, and −30°, is introduced to the MD simulation. The results are shown in Fig. 13 (plot at the same condition as the Fig. 7). When cutting with a positive rake angle, only small amount of atoms flow to the side of the tool edge forming small side flow on the machined surface. However, when cutting with negative rake angles, a large number of atoms flow to the side and increases with a decrease of negative rake angle. The number of atoms in the side flow region is counted and the results are displayed in Fig. 14. For all the three cutting directions, the number of side flow atoms decreases with an increase in positive rake angle.

### Effects of Inclination Angle

**Fig. 15:**
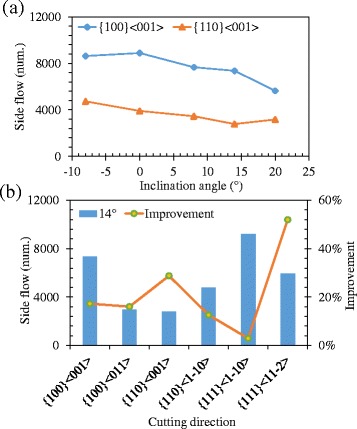
Number of atoms flow to the side of tool edge with different (**a**) inclination angles and (**b**) cutting directions

**Fig. 16:**
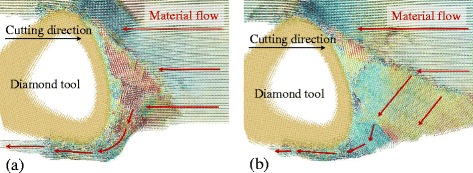
Displacement vector plots at inclination angle of (**a**) 14° and (**b**) 20° in cutting direction of {100} < 001>

In practice, the tool edge radius could not attain 0 nm and maintain sharp at cutting process. Therefore, the inclination angle seems to be a factor to suppress the side flow in nano-cutting. Figure 15a is the number of atoms flow to the side of tool edge with an inclination angle change from −8° to 20° in the cutting direction of {100} < 001 > and {110} < 001>. The results show that the number of side flow atoms decrease with an increase in inclination for both cutting directions. The displacement vector plots of the cutting processes with inclination angle of 14° nm and 20° nm are shown in Fig. 16 (at the same condition as the Fig. 7). Less material flows to the side of the tool edge forming the side flow on the machined surface. More simulations are performed with the inclination angle of 14° for all the six cutting directions. The results are displayed in Fig. 15b. Comparing the results in Fig. 6a which is the number of atoms flow to the side of cutting tool with inclination of 0°, the number of side flow atoms decreases for all the six cutting directions. The improvement for the cutting direction of {111} < 11-2 > is more than 50%. Therefore, the positive inclination angle would suppress the side flow in the nano cutting process and improve the machine surface quality.

## Conclusions

The effects of the crystallographic orientation and the cutting tool geometry, including tool edge radius, rake angle and inclination angle, on the side flow are investigated employing MD simulations. The conclusions can be drawn as follows:

1. The stagnation region in front of tool edge has been confirmed and is characterized by the stagnation radius *R*_*s*_ and stagnation height *h*_*s*_. The side flow is formed because the material at or under the stagnation region is extruded by the tool edge to flow to the side of the tool edge. The side flow is influenced by the position of stagnation region. Higher stagnation height would increase the size of the side flow and further deteriorate the machined surface quality.
2. The anisotropic nature of the material which partly determines the stagnation region also influence the side flow due to the different deformation mechanism under the action of the tool edge. At different cutting directions, the size of the side flow has great difference which would finally affect the machined surface quality. The cutting directions of {100} < 011>, {110} < 001>, and {110} < 1–10 > are beneficial to obtain a better surface quality.
3. The smaller feed decreases the size of the side flow, and no minimum PV value is found at which the PV value would increase when further decreases the feed. It is because the cutting distance used in this study is relatively small.
4. The side flow can be suppressed by optimizing the cutting tool geometry. Cutting tool with small edge radius, large positive rake angle, and inclination angle would decrease the side flow and improve the machined surface quality.
5. When the UCT is equal to or larger than the cutting tool edge, the material is removed in shearing mechanism. But when decreases the UCT, the material removal is dominated by extrusion mechanism.

## Abbreviations

MD: Molecular dynamics
UCT: Uncut chip thickness
